# MiR-150-5p inhibits cell proliferation and metastasis by targeting FTO in osteosarcoma

**DOI:** 10.32604/or.2024.047704

**Published:** 2024-10-16

**Authors:** LICHEN XU, PAN ZHANG, GUIQI ZHANG, ZHAOLIANG SHEN, XIZHUANG BAI

**Affiliations:** 1 Dalian Medical University, Dalian, 116044, China; 2 Department of Spinal Surgery, Dalian Municipal Central Hospital, Dalian, 116033, China; 3 Department of Orthopaedics, The People’s Hospital of China Medical University, People’s Hospital of Liaoning Province, Shenyang, 110016, China; 4 Department of Orthopedic, The Third Affiliated Hospital of Jinzhou Medical University, Jinzhou, 121000, China

**Keywords:** Fat mass and obesity associated (FTO), MiR-150-5p, Oosteosarcoma (OS), Cell proliferation, Cell metastasis, Exosome

## Abstract

**Background:**

Osteosarcoma (OS), recognized as the predominant malignant tumor originating from bones, necessitates an in-depth comprehension of its intrinsic mechanisms to pinpoint novel therapeutic targets and enhance treatment methodologies. The role of fat mass and obesity-associated (FTO) in OS, particularly its correlation with malignant traits, and the fundamental mechanism, remains to be elucidated.

**Materials and Methods:**

1. The FTO expression and survival rate in tumors were analyzed. 2. FTO in OS cell lines was quantified utilizing western blot and PCR. 3. FTO was upregulated and downregulated separately in MG63. 4. The impact of FTO on the proliferation and migration of OS cells was evaluated using CCK-8, colony formation, wound healing, and Transwell assays. 5. The expression of miR-150-5p in OS cells-derived exosomes was identified. 6. The binding of miR-150-5p to FTO was predicted by TargetScan and confirmed by luciferase reporter assay. 7. The impact of exosome miR-150-5p on the proliferation and migration of OS cells was investigated.

**Results:**

The expression of FTO was higher in OS tissues compared to normal tissues correlating with a worse survival rate. Furthermore, the downregulation of FTO significantly impeded the growth and metastasis of OS cells. Additionally, miR-150-5p, which was downregulated in both OS cells and their derived exosomes, was found to bind to the 3′-UTR of FTO through dual luciferase experiments. Exosomal miR-150-5p was found to decrease the expression of FTO and inhibit cell viability.

**Conclusions:**

We identified elevated levels of FTO in OS, which may be attributed to insufficient miR-150-5p levels in both the cells and exosomes. It suggests that the dysregulation of miR-150-5p and its interaction with FTO could potentially promote the development of OS.

## Introduction

Osteosarcoma (OS) is a malignant bone neoplasm predominantly impacting pediatric and adolescent populations [1,2]. While the amalgamation of surgical intervention and neoadjuvant chemotherapy has enhanced the five-year survival prognosis for newly diagnosed patients, considerable disparity in chemotherapeutic drug sensitivity continues to pose a substantial hindrance [3]. This impediment is markedly accentuated in patients manifesting metastasis or disease recurrence [4]. Consequently, the exploration of innovative targets from a mechanistic viewpoint becomes vital to comprehend the fundamental etiologies of metastasis and drug resistance.

The epitranscriptome, characterized by over 140 chemical modifications and reversible RNA transformations, plays a significant role in determining the fate of RNA [5,6]. The single nucleotide polymorphism (SNP) of FTO (fat mass- and obesity-associated protein) is intricately linked to obesity [7]. Despite the fact that FTO’s function as a demethylation enzyme was not clarified until 2010, its contribution to carcinogenesis has been progressively investigated in recent years [8–10]. FTO is pivotal in the transcription and translation of nucleic acids, upholding the stability of oncogene mRNA. It further stimulates metabolic reprogramming and immune evasion in neoplastic cells [11–13]. Inhibition of FTO obstructs cell propagation and migration in acute myeloid leukemia [14], glioma [15], and breast cancer [16]. Conversely, FTO serves as a tumor suppressor in ovarian cancer [17]. Nonetheless, the role of FTO, potentially linked to a malignant phenotype, and the underlying mechanism remains to be fully elucidated.

The anti-tumor agent, miR-150-5p, has been observed to exhibit diminished expression across a spectrum of tumor types [18,19]. Its specific influence on OS progression and cisplatin sensitivity, however, remains unexplored. Prior research has indicated that exosomes can alter tumor sensitivity to chemotherapeutic agents through the conveyance of microRNAs, a process which mitigates side effects and augments microRNA concentration within recipient cells [20–22]. Despite these findings, the impact of exosome-mediated microRNA delivery on the malignancy attributes of tumors necessitates additional scrutiny.

This study investigates the impact of FTO on the proliferation and metastasis of OS. Identified as the target gene of miR-150-5p, FTO’s influence is significant. Furthermore, exosomal-miR-150-5p has the potential to suppress cell proliferation and migration within OS.

## Materials and Methods

### Bioinformatic analysis

The expression patterns of FTO and miR-150-5p were scrutinized utilizing the GEPIA and ENCORI databases. Concurrently, the survival rate was also analysed.

### OS tissue microarray FTO detection

The expression of FTO in OS was ascertained using tissue microarrays procured from Bioaitech (L714901, Xi’an, China). Subsequently, a standard Immunohistochemistry (IHC) analysis was conducted on the FTO present in these OS microarrays.

### Cell culture

The osteoblast cell line hFOB1.19, along with Human OS lines MG-63, HOS, U-2OS, and SAOS-2, were obtained from Procell (Wuhan, China). STR identification of cell lines used in this study can be viewed in the website of Procell (https://www.procell.com.cn). MG-63, HOS, and hFOB1.19 cell lines were propagated in a nutrient-rich medium supplemented with high glucose-DMEM. In contrast, the U-2OS and SAOS-2 cell lines were cultivated in Minimum Essential Medium (MEM) fortified with a 10% concentration of Fetal Bovine Serum (FBS) (Gibco; Thermo Fisher Scientific, Inc., NY, USA). The cells underwent cultivation within a humidified environment, maintained at a constant temperature of 37°C, under a 5% concentration of CO_2_.

### Lentivirus

The lentivirus overexpressing FTO was engineered by GENE (Shanghai, China). The lentiviruses encapsulating FTO shRNA, encompassing the target sequences sh1: 5′-TCACCAAGGAGACTGCTATTT-3′ and sh2: 5′-CTAGGGTTTTGCTTCCAGAATT-3′, in addition to the negative control (shNC), were procured from HANBIO (Shanghai, China).

### RNA extraction and quantitative real-time PCR analysis

The Total RNA was isolated utilizing the TRIzol reagent (Invitrogen, NY, USA), in accordance with the manufacturer’s guidelines. This was followed by the synthesis of cDNA, executed using the PrimeScript RT Reagent Kit (Takara, Dalian, China). The qRT-PCR analysis was conducted utilizing the Roche LightCycler96 system (Roche, Basel, Switzerland) in combination with the SYBR Green SuperMix. To maintain precision in the results, β-actin and U6 were judiciously chosen as internal standard references. The quantification of relative gene expression was ascertained utilizing the 2^−ΔΔCt^ method. The primer sequences can be found in Table 1.

**Table 1:** The primers used in qRT-PCR

Gene	Primer	Sequence
FTO	Forward	5′-AGACACCTGGTTTGGCGATA-3′
	Reverse	5′-CCAAGGTTCCTGTTGAGCAC-3′
β-actin	Forward	5′-GGCGGCACCACCATGTACCCT-3′
	Reverse	5′-AGGGGCCGGACTCGTCATACT-3′
miR-150-5p	Forward	5′-ACTGTCTCCCAACCCTTGTA-3′
	Reverse	5′-GTGCAGGGTCCGAGGT-3′
U6	Forward	5′-CTCGCTTCGGCAGCACA-3′
	Reverse	5′-AACGCTTCACGAATTTGCGT-3′

### Western blot

The cells underwent lysis via the application of RIPA buffer (Beyotime Biotech, Shanghai, China). Subsequently, protein quantification ensued, utilizing a BCA assay Kit (Pierce, Thermo Fisher Scientific, Inc., NY, USA). The lysates subsequently underwent a 10% SDS-PAGE procedure, followed by a transfer onto polyvinylidene fluoride (PVDF) membranes (Millipore, Merck, Darmstadt, Germany). Subsequently, the membranes were treated with 5% non-fat milk to block non-specific binding sites, and then incubated at a temperature of 4°C overnight with the primary antibody. Subsequently, a secondary antibody was introduced and allowed to incubate at ambient temperature for a duration of one hour. Dilution ratio: primary antibody 1:1000, secondary antibody 1:5000. anti-FTO antibody (Abcam#ab126605), anti-GAPDH antibody (Cell signaling Technology #5174), anti-CD9 antibody (Proteintech#20597-1-AP), anti-CD63 antibody (Proteintech# 25682-1-AP), anti-TSG101 antibody (Proteintech# 28283-1-AP), HRP-goat-anti-rabbit IgG (Proteintech#SA00001-2). Visualization of protein bands was achieved through the application of enhanced chemiluminescence (ECL, Pierce, Thermo Fisher Scientific, Inc., NY, USA).

### Cell viability assay

A total of 2 × 10^3^ cells were cultured in each well of a 96-well plate. Upon assessment, a tenth of the volume of CCK-8 reagent (Dojindo, Japan) was integrated into every well. Following a two-hour incubation period, the absorbance was quantified at 490 nm. As a result, a growth curve based on “absorbance-time” was generated.

A single-cell suspension comprising 400 cells was seeded into six-well plates. These cells were subsequently cultured under standard conditions for a duration spanning 10 to 14 days, with the culture medium being refreshed tri-weekly. Upon the establishment of over 50 cellular clones, the cells underwent a gentle wash with Phosphate-Buffered Saline (PBS), followed by fixation with 4% paraformaldehyde at ambient temperature for a 20-min period. The cells were then subjected to staining using a 0.1% crystal violet solution, a process that lasted between 5 to 10 min, and were subsequently rinsed with water. The clone formation was subsequently observed, documented through photography, and quantified.

### Wound healing assay

Upon attaining a cell density of 70%–80% within the six-well plate, a 200 μL pipette tip was employed to inflict controlled wounds. Following this, any detached cells were meticulously removed via a sterile Phosphate-Buffered Saline (PBS) rinse. The cells were subsequently cultivated in a serum-deprived medium for a period of 48 h. Utilizing a microscope, images of the inflicted wound were captured at the initial time point (0 h) and after the 48-h incubation period. The residual wound area was quantified in comparison to its original size at the 0-h mark.

### Transwell assay

In the upper compartment of the Transwell chamber (Corning, USA), 5000 cells were introduced in a volume of 200 μL of serum-free medium. The lower compartment was supplemented with 600 μL of medium, serving as a cellular stimulant. Following a 24-h incubation period, the cells residing in the upper compartment were delicately removed using a cotton swab. The cells that had successfully migrated through the membrane were stabilized with paraformaldehyde, followed by a thorough rinse with water. For visualization purposes, the cells were stained using a 0.1% crystal violet solution. Finally, the cells were examined, imaged, and quantified in four arbitrarily selected visual fields under a microscope.

### Exosome extraction and detection

The cell supernatant underwent a sequential centrifugation process, initially at 200–300 *g* for a 10-min duration, followed by an increased speed of 3000 *g* for another 10 min, and finally culminating at 10,000 *g* for 30 min. This procedure facilitated the concentration of exosomes within a 100 kD ultrafiltration apparatus.

Subsequently, the sample was deposited onto a copper mesh integrated with a membrane. The suspension’s concentration was meticulously adjusted, followed by the addition of a phosphotungstic acid-based staining solution for a period of 3–5 min. Any surplus dye was absorbed using a filter paper, and the sample was then desiccated prior to examination under a transmission electron microscope. The Nanoparticle Tracking Analysis (NTA) was conducted by Dlmbiotech (Wuhan, China).

### Statistical analysis

The data are represented as the mean ± standard deviation. To compare the data across different groups, we employed the Student’s *t*-test. A *p*-value of less than 0.05 was deemed to indicate statistical significance. GraphPad Prism 8 was utilized for chart creation, while ImageJ V1.8.0 was used for the analysis of Western blot results.

## Results

### The overexpression of FTO is correlated with unfavorable prognostic outcomes in multiple neoplasms, inclusive of OS

This study primarily concentrates on exploring the expression and functionality of FTO within tumor contexts. Initially, we conducted an analysis of FTO’s expression across various cancers utilizing the GEPIA database. It was observed that the GEPIA database contains a limited number of OS samples, providing data predominantly for sarcomas. Given the scarcity of research concerning FTO’s involvement in OS, our subsequent investigation focused on elucidating the role of FTO in this specific type of cancer.

**Figure 1:**
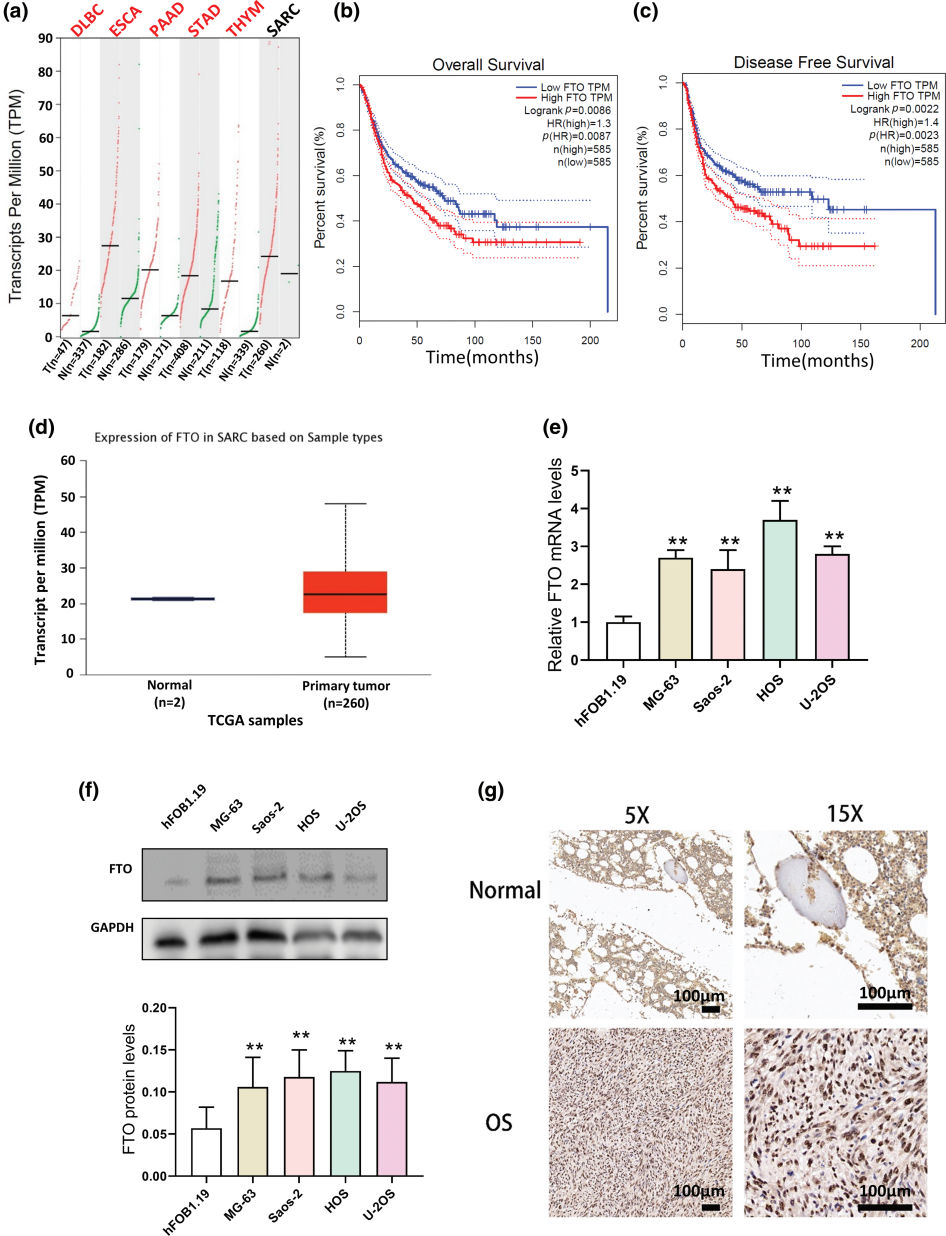
Expression of FTO in various tumors in GEPIA database and its relationship with prognosis. (a) Expression of FTO in DLBC, ESCA, PAAD, STAD, THYM, and SARC in GEPIA database. (b, c) Expression of FTO and analysis of survival in patients of tumor patients by GEPIA database. (d) Expression of FTO in SARC by TCGA database. (e) mRNA levels of FTO in OS cells and osteoblasts detected by PCR. (f) Protein levels of FTO in OS cells and osteoblasts analysed by western blot. (g) FTO were detected by IHC in OS tissue chip. **p < 0.01, scale bar = 100 μm.

As GEPIA and TCGA database indicates, FTO is upregulated in various tumors, including sarcoma (SARC) (Fig. 1a,d). Higher FTO levels correlates with poor survival rates (Fig. 1b,c). Further, PCR, Western blot and IHC analyses demonstrate a significant elevation in FTO expression in OS cells and human OS tissues (Fig. 1e–g). This compelling evidence strongly supports the crucial role of FTO in the pathogenesis of OS.

### FTO is a crucial factor in the proliferation of OS

**Figure 2:**
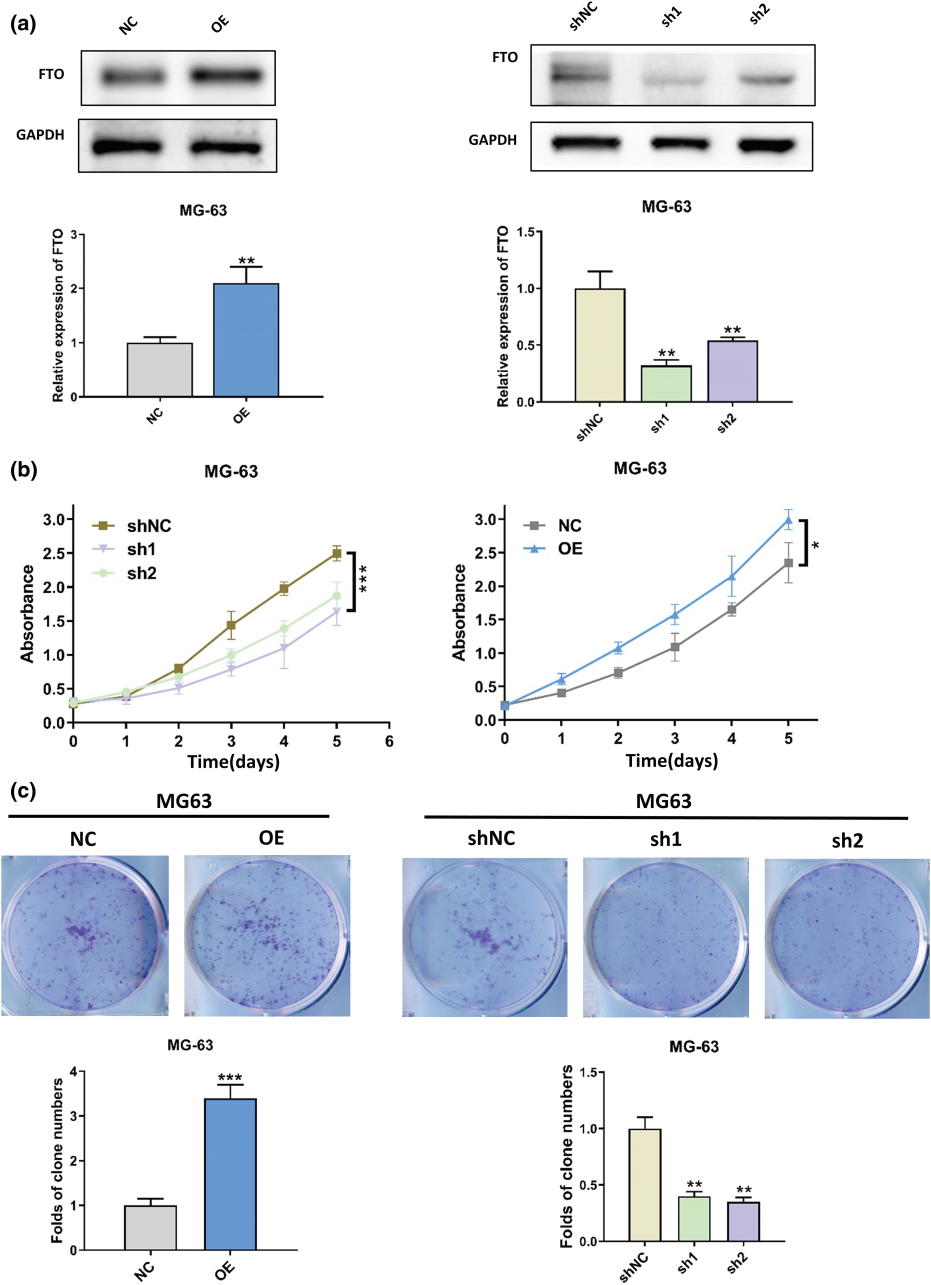
Effect of FTO on the proliferation of OS cells. (a) Stable overexpression/knockdown FTO identification confirmed by western blot. (b) CCK-8 assay was used to detect the effect of FTO overexpression/knockdown on cell viability. (c) Colony formation assay of FTO regulated MG-63. *p < 0.05, **p < 0.01, ***p < 0.005.

Lentivirus was used to establish MG-63 cells with stable FTO overexpression or knockdown, as shown in Fig. 2a. The CCK-8 assay demonstrated that FTO overexpression augmented cell viability, whereas FTO knockdown elicited a contrary effect, as depicted in Fig. 2b. The colony formation assay further confirmed the proliferative role of FTO within cells, as demonstrated in Fig. 2c.

### FTO is essential for the migration of OS

**Figure 3:**
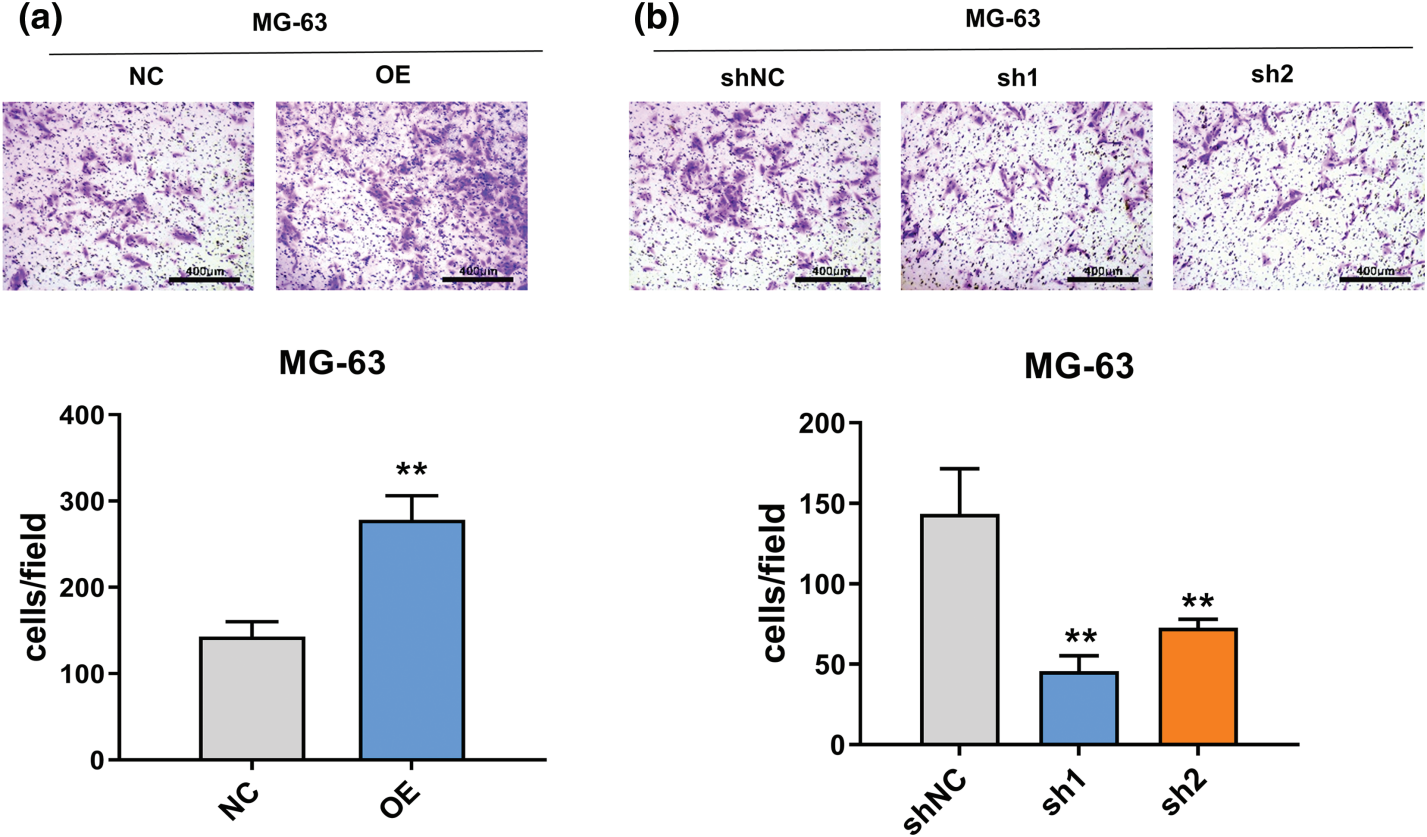
Effect of FTO on the migration of OS cells. (a, b) Transwell assay was used to detect the effect of FTO overexpression (a)/knockdown (b) on cell invasion ability. **p < 0.01, scale bar = 400 μm.

**Figure A1:**
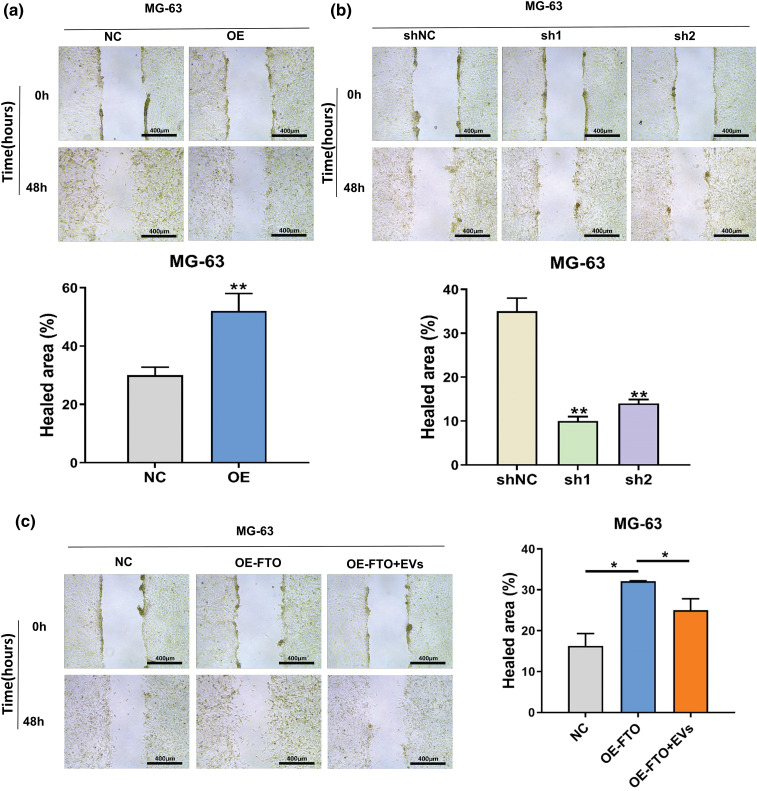
Wound healing assay of MG-63 after FTO overexpression (a)/knockdown (b) FTO, and exosome miR-150-5p treated (c). *p < 0.05; **p < 0.01.

The wound healing assay results, as depicted in Fig. A1a, underscore the pivotal role of FTO in augmenting cell migration. In contrast, as Fig. A1b illustrates, FTO inhibition significantly impairs the migratory capabilities of cells. By the way, it is important to mention that the cells showed limited motility in the context of wound healing. Furthermore, the Transwell assay findings, represented in Fig. 3a, indicate that FTO overexpression precipitates a marked surge in cell invasion. Conversely, FTO knockdown culminates in a significant curtailment of cell invasion, as evidenced in Fig. 3b. These observations underscore the integral role of FTO in modulating cell migration and invasion.

### MiR-150-5p is down-regulated in OS cells derived exosomes derived from OS cells

**Figure 4:**
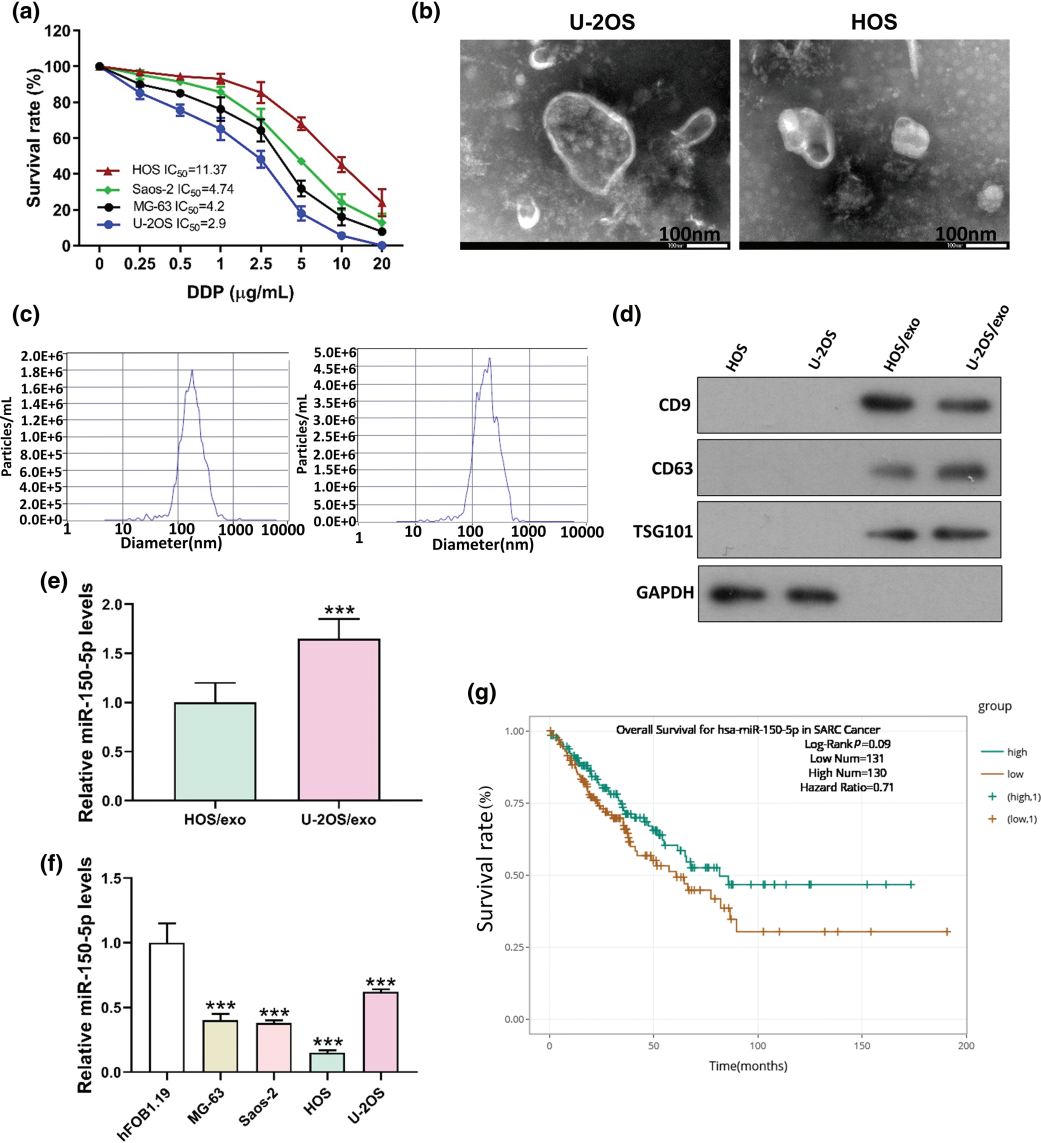
miR-150-5p is down-regulated in exosomes derived from OS cells. (a) IC50 of DDP of different OS cells was detected by CCK8. (b) TEM of HOS, U-2OS supernatant exosome. (c) HOS, U-2OS supernatant exosome NTA detection. (d) Western blot detection of exosome markers in HOS and U-2OS supernatant. (e) Real-time PCR detection of miR-150-5p in exosomes of HOS and U-2OS supernatant. (f) Real-time PCR analysis of miR-150-5p levels in human osteoblasts hFOB1.19 and OS cells (MG-63, Saos-2, HOS, U-2OS). (g) Analysis of miR-150-5p levels and survival in patients with sarcoma in ENCORI database. ***p < 0.005, scale bar = 100 nm.

Exosomes from the supernatant of U-2OS cells, characterized by high cisplatin sensitivity (IC50 = 2.9 μg/mL), and HOS cells, known for their low cisplatin sensitivity (IC50 = 11.37 μg/mL), were isolated (Fig. 4a–d). A comprehensive analysis incorporating the TargetScan database and the evaluation of differentially expressed miRNAs in the exosomes of these two cell strains revealed a notable disparity in the concentration of miR-150-5p. This microRNA, a potential upstream regulator of FTO, exhibited different levels in the exosomes derived from the supernatant of the two cell cultures (Fig. 4e). Moreover, the concentration of miR-150-5p in OS cells was significantly diminished compared to that in osteoblasts (Fig. 4f). This observation suggests a potential role of miR-150-5p downregulation in OS pathogenesis. In alignment with this, the ENCORI database analysis indicated that sarcoma patients expressing high levels of miR-150-5p demonstrated improved survival rates (Fig. 4g).

### MiR-150-5p targets FTO

**Figure 5:**
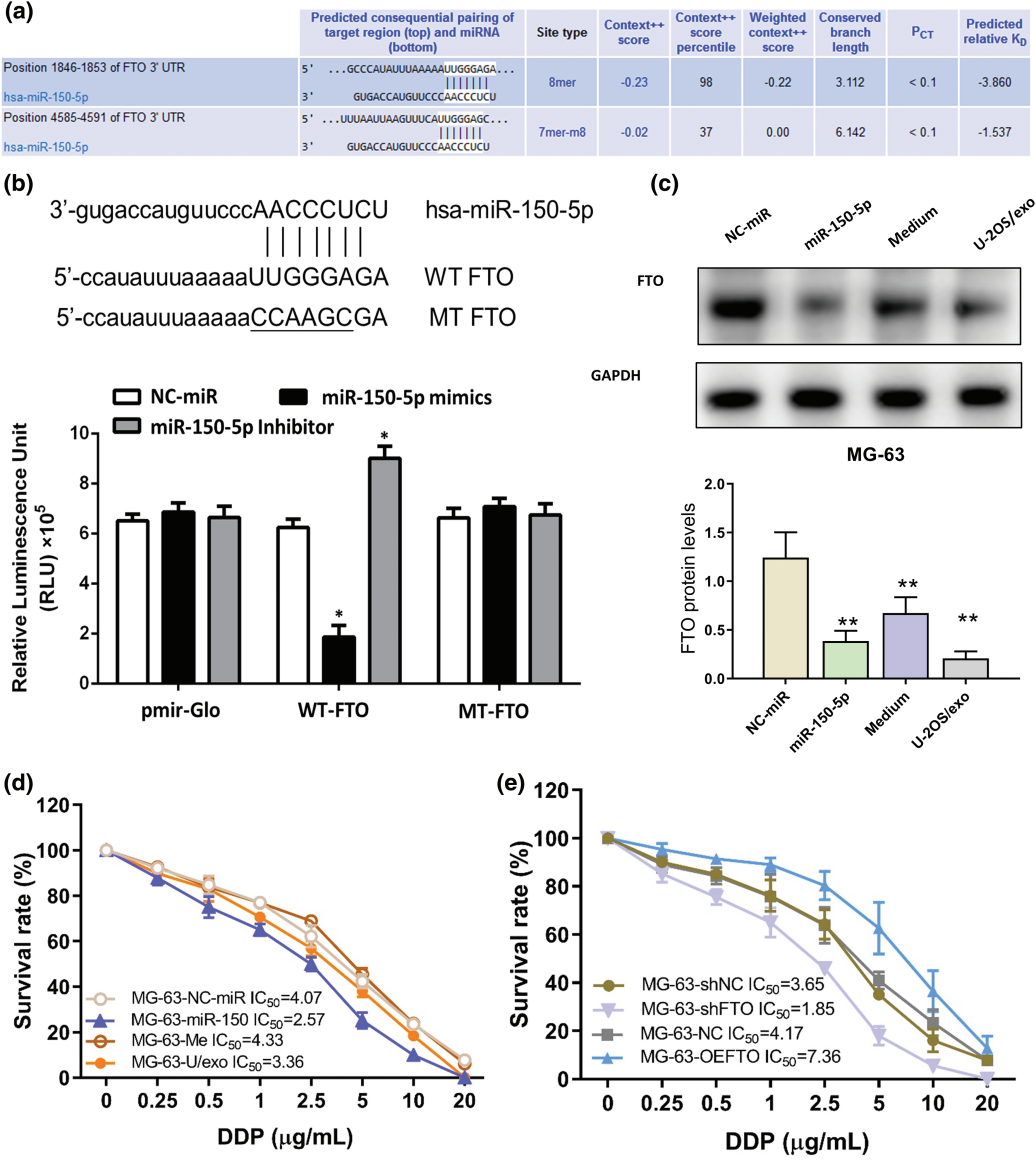
FTO is the target gene of miR-150-5p. (a) TargetScan predicts the binding site of miR-150-5p to FTO. (b) luciferase reporter assay of miR-150-5p and FTO. (c) The protein levels of FTO within MG-63 cells are detected by western blot after miR-150-5p overexpression, in conjunction with the co-incubation of U-2OS supernatant-derived exosomes. (d) Effects of overexpression of miR-150-5p or co-incubation with exosomes of U-2OS supernatant on sensitivity to cisplatin of MG-63 cells. (e) Effects of overexpression or knockdown of FTO on sensitivity to cisplatin of MG-63 cells. *p < 0.05, **p < 0.01.

The binding site of miR-150-5p within the 3′ untranslated region (UTR) of FTO has been accurately predicted by TargetScan, as depicted in Fig. 5a. This prediction was subsequently corroborated through a luciferase reporter gene assay. This assay revealed the interaction between miR-150-5p and the wild-type (WT) FTO 3′UTR, while no such interaction was discernible with the mutated (MT) FTO, which possesses modified binding sites, as illustrated in Fig. 5b. Furthermore, when MG-63 cells were co-cultured with miR-150-5p and U-2OS supernatant exosomes, a significant reduction in FTO levels was observed, as demonstrated in Fig. 5c.

The role of the miR-150-5p/FTO axis in influencing the cisplatin sensitivity of OS cells was investigated using a CCK-8 assay on MG-63 cells. These cells were subjected to overexpression of miR-150-5p, co-incubation with exosomes, or FTO modulation. The findings indicated that both miR-150-5p overexpression and FTO interference enhanced the cisplatin sensitivity of the OS cells (Figs. 5d,e). Additionally, the cisplatin sensitivity of OS cells was found to be augmented by exosomes from U-2OS supernatant, albeit to a lesser extent than miR-150-5p overexpression (Fig. 5d). This suggests the potential involvement of other molecular entities within exosomes in modulating cisplatin sensitivity. Conversely, FTO overexpression was observed to enhance the cisplatin tolerance of OS cells (Fig. 5e).

### Exosomes loaded with miR-150-5p can reduce the cancer-promoting function of FTO

**Figure 6:**
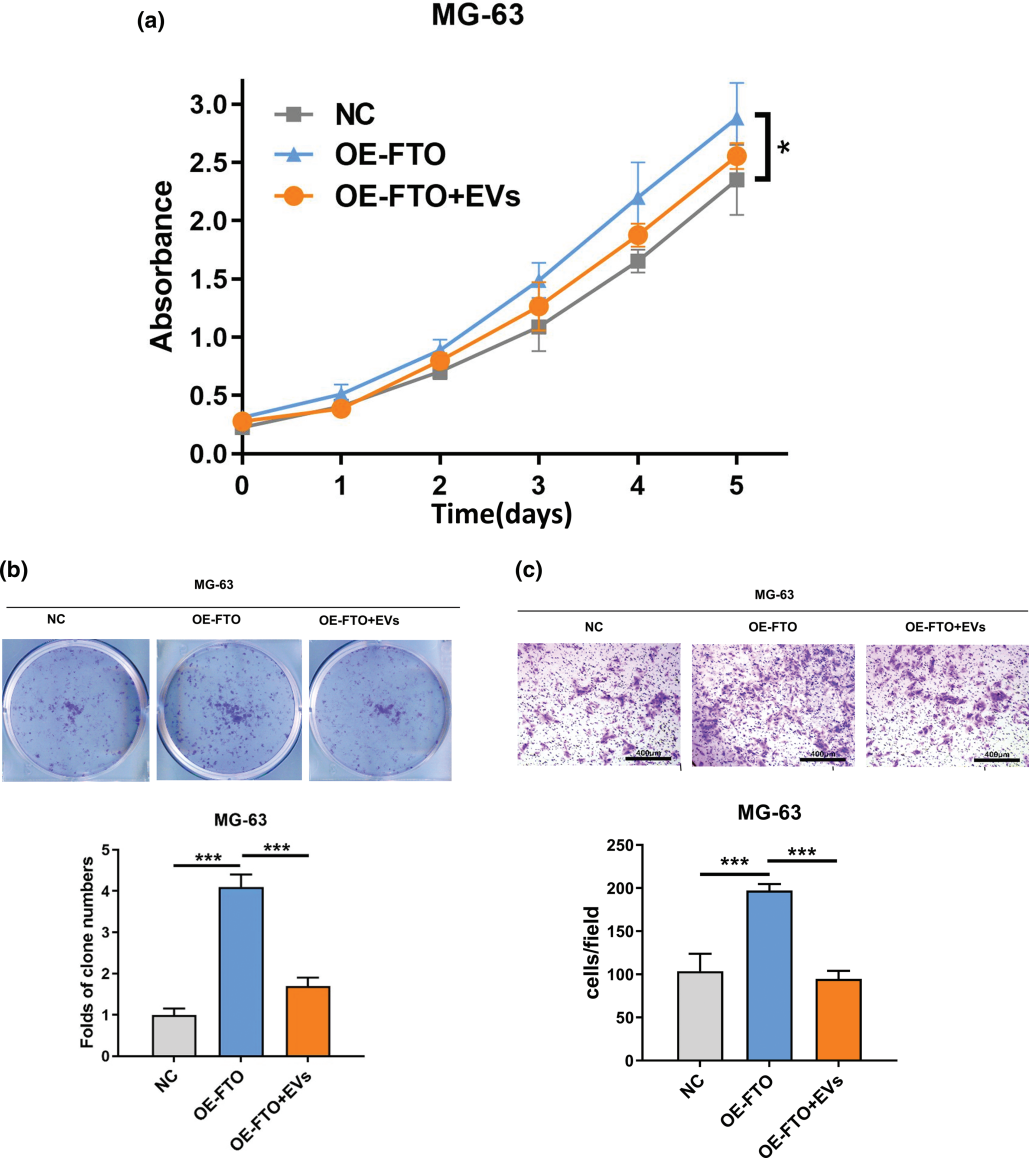
Effects of exosome miR-150-5p on proliferation and migration of OS cells overexpressing FTO. (a) CCK-8 assay. (b) Colony formation. (c) Transwell assay. *p < 0.05, ***p < 0.005, scale bar = 400 μm.

Exosomal miR-150-5p (Evs) has been observed to attenuate the proliferative impact instigated by the overexpression of FTO in OS cells (Fig. 6a,b). Furthermore, the cellular migration of OS cells, characterized by FTO overexpression, was notably reduced following a 48-h co-incubation period with exosomes enriched with miR-150-5p (Figs. 6c and A1c).

## Discussion

Since its identification as the inaugural mRNA demethylation enzyme of m6A in 2010, the significance of m6A in metabolism, biological processes, and pathogenesis has progressively attracted scholarly interest. In a preponderance of cancer variants, FTO exhibits high expression, thereby fostering cancer progression. Conversely, in certain specific tumor categories, it functions as a tumor suppressor. Nevertheless, it is crucial to acknowledge that there is no consensus regarding the expression pattern and pathological role of FTO in some tumor types. This incongruity could be ascribed to elements such as the pronounced heterogeneity of cancer specimens, the classification of sample sizes, and the diversity in detection methodologies.

Significantly, FTO continues to foster the progression of lung cancer [23], bladder cancer [24], and thyroid cancer [25], despite its down-regulation at the RNA level. Posttranslational modifications, including the elevation of FTO protein levels in bladder cancer via USP18 deubiquitination [24], may also serve a pivotal function in the pathophysiological processes mediated by FTO. For the effective direction of therapeutic interventions, a thorough comprehension of the FTO’s role in various cancer types or subtypes is paramount. Predominantly, FTO operates as an m6A demethylase in most research, modulating the expression of its target molecules post-transcriptionally. Nevertheless, the processes through which m6A dictates RNA destiny remain predominantly elusive, notwithstanding prior assertions that m6A mRNA exhibits greater stability compared to other mRNAs [26].

AML cells that exhibit elevated FTO expression are dependent on FTO-mediated m6A demethylation for the degradation of tumor suppressor gene transcripts. This mechanism enhances the stability of oncogenes, notably MYC, and metabolic regulatory genes, including PFKP and LDHB. Consequently, this process facilitates the proliferation and differentiation of these cells [27,28]. At present, there is no unified understanding regarding the expression and function of FTO in Non-Small Cell Lung Cancer (NSCLC). Certain research suggests that Lung Squamous Cell Carcinoma (LUSC) patients with elevated FTO expression exhibit a lower survival rate, a trend not observed in Lung Adenocarcinoma (LUAD) patients [29]. Conversely, other studies argue that FTO expression does not impact LUSC patient survival, but rather enhances the survival rate of LUAD patients with high FTO expression [23]. Moreover, FTO has been shown to stimulate the proliferation of various NSCLC cell lines from both subtypes. The suppression of FTO hinders the proliferation and migration of numerous LUSC cell lines, while the overexpression of FTO, as opposed to the catalysis of inactive FTO mutants, induces contrasting phenotypes.

Mechanically, FTO has been demonstrated to stabilize the mRNA of myeloid zinc finger 1 (MZF1) through demethylation. This action has been shown to induce MYC transcription, thereby promoting the occurrence of LUAD [30]. Upon the suppression of FTO, the mRNA of Ubiquitin-Specific Peptidase 7 (USP7) experiences hypermethylation, which subsequently results in a decrease in its transcription level [31]. Although the overexpression of USP7 can rectify the growth anomalies induced by FTO suppression, the underlying molecular mechanism remains elusive. In Luminal A and triple-negative breast cancer cell lines, FTO suppression fosters apoptosis and impedes cell migration, both *in vitro* and *in vivo* [32]. Beyond its function in modulating cancer cell proliferation, metastasis, and CSC self-renewal, FTO also plays a pivotal role in the regulation of the tumor microenvironment (TME) and tumor metabolism. It has been found to be associated with glycolysis and glutamine uptake [28,33].

Given the complex nature of tumor immune evasion and the diversity of both the host and the tumor, monotherapy often falls short in completely eliminating tumors. As such, combination therapy emerges as a more advantageous strategy. For example, the application of IDH inhibitors and FTO inhibitors has demonstrated efficacy in treating patients with IDH mutant cancers. In situations where FTO is significantly overexpressed in AML patients with FLT3-ITD, a therapeutic approach combining FTO inhibitors and FLT3 inhibitors can be implemented. Moreover, the introduction of demethylating agents, such as 5-Aza and decitabine (typically employed as primary treatments in elderly AML patients), markedly amplifies FTO signaling in AML cells [11]. Studies have substantiated that the induction of apoptosis in neoplastic cells can be achieved through the use of FTO inhibitors. The escalation in neoplastic cell apoptosis, facilitated by the suppression of FTO via pharmacological intervention, could potentially unveil tumor neoantigens. This process may subsequently stimulate immunogenic cell death and enhance the tumor’s sensitivity to immunotherapy. Furthermore, the strategic targeting of FTO has the potential to attenuate the expression of immune checkpoint genes, such as PD-L1 and LILRB4, within neoplastic cells [11,34]. The cooperative effect of the FTO inhibitor Dac51 and the anti-PD-L1 blocker *in vivo* has laid a theoretical groundwork for this combined therapeutic approach [33].

Consistent with the previous study by Tsuruta et al. [34] and Lv et al. [35], our investigation further corroborates the role of FTO in augmenting the proliferation and migration of OS cells. Conversely, the suppression of FTO results in an inverse effect. Preliminary studies have suggested that FTO may escalate the progression of OS via KLF3 and DACT1 pathways. Nevertheless, the causative factors behind the heightened presence of FTO in OS remain elusive, thus necessitating our exploration.

Research has substantiated that the suppression of miR-21 impedes the advancement of OS (OS), underscoring its potential as a therapeutic target and its prospective utility as an OS biomarker [36]. He et al. have disclosed the participation of the miR-34 cluster in the genesis of OS [37]. Sun et al. have clarified the correlation between miR-143 and FOSL2, a pivotal regulatory element in bone development, within the context of OS. They noted a diminished expression of miR-143 in OS tissue relative to normal bone tissue, and established that miR-143 curbs the proliferation, migration, and invasion of OS by curtailing FOSL2 expression [38]. Jones et al. ascertained that the expression of miRNA mirrors the pathogenesis and malignancy in patients afflicted with OS [39]. Contemporary studies have unveiled a down-regulation of miR-16 and miR-665 in OS cell lines (MG63, U2OS) and OS specimens [40], signifying their capacity to restrain tumor cell progression and invasion via the modulation of RAB23 [41,42]. Signifying their capacity to restrain tumor cell progression and invasion via the modulation of RAB23.

Exosomes derived from tumors are microvesicles, characterized by their cup-like morphology and dimensions ranging between 30 to 100 nm [43–45]. These vesicles primarily encapsulate a diverse array of genetic messengers, encompassing DNA, mRNA, miRNA, cytoplasmic proteins, and lipids [46–48]. Studies have indicated that these exosomes can alter tumor susceptibility to chemotherapeutic agents via the microRNAs they transport. Moreover, the conveyance of microRNAs through exosomes can mitigate the adverse effects linked with direct administration, thereby enhancing microRNA levels in recipient cells.

A study conducted by Jerez et al. involved a genetic examination of the predicted targets of miRNAs in extracellular vesicles derived from OS. The findings suggested that miRNAs originating from OS cell lines could potentially modulate the metastatic capability of tumor cells by suppressing gene networks associated with apoptosis and cell adhesion [49]. Nevertheless, a comprehensive exploration of the influence of exosome-encapsulated microRNAs on tumor-associated malignant phenotypes remains to be conducted.

While the regulatory mechanisms governing miRNA encapsulation during oncogenic progression exhibit significant variability, this investigation demonstrates a decrease in miR-150-5p expression within both extracellular vesicles (EVs) and neoplastic tissues. This underscores the potential value of miR-150-5p as a predictive biomarker for patient responsiveness to cisplatin. Existing research substantiates the critical role of miR-150-5p in facilitating the progression of diverse malignancies, with particular emphasis on lung cancer. Hypoxic conditions precipitate an elevation in miR-150-5p concentrations within EVs, concurrent with a reduction in CD226 expression within natural killer (NK) cells. NK cells excrete S100A8 and other immunosuppressive agents, thereby catalyzing metastasis and the genesis of pulmonary tumor nodules. Conversely, the downregulation of miR-150-5p instigates a decrease in oncogenic development and a resurgence of CD226 expression, thereby presenting a potential therapeutic strategy [50]. In colorectal carcinoma, the activation of Wnt signaling stimulates miR-150-5p, which subsequently targets CREB. This interaction results in the downregulation of E-cadherin and ZO-1, thereby fostering epithelial-mesenchymal transition (EMT) and augmenting cellular invasion and migration. Furthermore, miR-150-5p has been demonstrated to play a significant role in the metastatic progression of non-small cell lung cancer by targeting FOXO4. The overexpression of miR-150-5p amplifies migration and EMT [51].

Patients diagnosed with colorectal cancer exhibit diminished levels of exosome miR-150-5p in comparison to their healthy counterparts, indicating the potential of miR-150-5p to serve as a biomarker for colorectal cancer detection [52]. Contrarily, another colorectal cancer research revealed that the suppression of miR-150-5p resulted in augmented cell viability and an upsurge in β-catenin expression within SW480 and HT-29 cells, thereby fostering colony formation. This evidence implies that miR-150-5p might function as a tumor suppressor in colorectal cancer [53]. MicroRNAs (miRNAs) have been established as potential biomarkers. In this study, we have successfully validated the practicability of identifying tissue-expressed miRNAs, not in plasma, but in extracellular vesicles, thus presenting the possibility of obviating the necessity for invasive biopsies. The significance of extracellular vesicle miRNAs (EV-miRNAs) in other types of cancer has also been substantiated. A key limitation of this study is the incomplete understanding of the mechanisms driving miRNA expression changes in exosomes and the downstream pathways modulated by FTO as a demethylase. Further research is needed to unravel the specific triggers and regulatory networks influencing exosomal miRNA expression, as well as to identify and characterize the downstream targets and signaling cascades of FTO. Addressing these limitations could advance our knowledge and lead to the development of novel diagnostic markers and therapeutic strategies.

In this research, we focused on microRNA-miR-150-5p present in the exosomes derived from the supernatant of OS cells exhibiting diverse cisplatin sensitivity. Through the analysis of biological informatics data, we discerned a substantial disparity in the concentration of miR-150-5p between normal osteoblasts and OS cells. Additionally, we verified that FTO is a direct target of miR-150-5p. The primary objective of our study was to explore the influence of exosomal miR-150-5p on the proliferation and migration of OS cells.

## Conclusions

The overexpression of FTO, a crucial player in the proliferation and migration of OS cells, has been linked to unfavorable prognoses in a variety of tumors, including OS. Conversely, exosomal-miR-150-5p serves as a mitigating factor in OS progression, exerting its effect by specifically targeting FTO.

## Data Availability

The data and materials used in this study are available upon reasonable request from the corresponding author.

## References

[1] Dieffenbach BV, Murphy AJ, Liu Q, Ramsey DC, Geiger EJ, Diller LR, et al. Cumulative burden of late, major surgical intervention in survivors of childhood cancer: a report from the childhood cancer survivor study (CCSS) cohort. Lancet Oncol. 2023;24(6):691–700. doi:10.1016/S1470-2045(23)00154-7; 37182536 PMC10348667

[2] Beird HC, Bielack SS, Flanagan AM, Gill J, Heymann D, Janeway KA, et al. Osteosarcoma. Nat Rev Dis Primers. 2022;8(1):77. doi:10.1038/s41572-022-00409-y; 36481668

[3] Jiang Y, Wang J, Sun M, Zuo D, Wang H, Shen J, et al. Multi-omics analysis identifies osteosarcoma subtypes with distinct prognosis indicating stratified treatment. Nat Commun. 2022;13(1):7207. doi:10.1038/s41467-022-34689-5; 36418292 PMC9684515

[4] Yang X, Gao S, Yang B, Yang Z, Lou F, Huang P, et al. Bioinspired tumor-targeting and biomarker-activatable cell-material interfacing system enhances osteosarcoma treatment via biomineralization. Adv Sci. 2023;10(22):e2302272. doi:10.1002/advs.202302272; 37211693 PMC10401161

[5] Venkataramany AS, Schieffer KM, Lee K, Cottrell CE, Wang PY, Mardis ER, et al. Alternative RNA splicing defects in pediatric cancers: new insights in tumorigenesis and potential therapeutic vulnerabilities. Ann Oncol. 2022;33(6):578–92. doi:10.1016/j.annonc.2022.03.011; 35339647 PMC12361925

[6] Li HB, Huang G, Tu J, Lv DM, Jin QL, Chen JK, et al. METTL14-mediated epitranscriptome modification of MN1 mRNA promote tumorigenicity and all-trans-retinoic acid resistance in osteosarcoma. eBioMedicine. 2022;82:104142. doi:10.1016/j.ebiom.2022.104142; 35810559 PMC9272358

[7] Speakman JR. FTO effect on energy demand versus food intake. Nature. 2010;464(7289):E1. doi:10.1038/nature08807; 20360686

[8] Zuidhof HR, Calkhoven CF. Oncogenic and tumor-suppressive functions of the RNA demethylase FTO. Cancer Res. 2022;82(12):2201–12. doi:10.1158/0008-5472.CAN-21-3710; 35303057

[9] Zhang J, Wei J, Sun R, Sheng H, Yin K, Pan Y, et al. A lncRNA from the FTO locus acts as a suppressor of the m6A writer complex and p53 tumor suppression signaling. Mol Cell. 2023;83(15):2692–708. doi:10.1016/j.molcel.2023.06.024; 37478845 PMC10427207

[10] Li Y, Su R, Deng X, Chen Y, Chen J. FTO in cancer: functions, molecular mechanisms, and therapeutic implications. Trends Cancer. 2022;8(7):598–614. doi:10.1016/j.trecan.2022.02.010; 35346615

[11] Su R, Dong L, Li Y, Gao M, Han L, Wunderlich M, et al. Targeting FTO suppresses cancer stem cell maintenance and immune evasion. Cancer Cell. 2020;38(1):79–96.e11. doi:10.1016/j.ccell.2020.04.017; 32531268 PMC7363590

[12] Huang Y, Su R, Sheng Y, Dong L, Dong Z, Xu H, et al. Small-molecule targeting of oncogenic FTO demethylase in acute myeloid leukemia. Cancer Cell. 2019;35(4):677–91.e10. doi:10.1016/j.ccell.2019.03.006; 30991027 PMC6812656

[13] Liu L, Gu M, Ma J, Wang Y, Li M, Wang H, et al. CircGPR137B/miR-4739/FTO feedback loop suppresses tumorigenesis and metastasis of hepatocellular carcinoma. Mol Cancer. 2022;21(1):149. doi:10.1186/s12943-022-01619-4; 35858900 PMC9297645

[14] Huang Y, Xia W, Dong Z, Yang CG. Chemical inhibitors targeting the oncogenic m6A modifying proteins. Acc Chem Res. 2023;56(21):3010–22. doi:10.1021/acs.accounts.3c00451; 37889223

[15] Dai W, Tian R, Yu L, Bian S, Chen Y, Yin B, et al. Overcoming therapeutic resistance in oncolytic herpes virotherapy by targeting IGF2BP3-induced NETosis in malignant glioma. Nat Commun. 2024;15(1):131. doi:10.1038/s41467-023-44576-2; 38167409 PMC10762148

[16] Chen F, Song C, Meng F, Zhu Y, Chen X, Fang X, et al. 5′-tRF-GlyGCC promotes breast cancer metastasis by increasing fat mass and obesity-associated protein demethylase activity. Int J Biol Macromol. 2023;226:397–409. doi:10.1016/j.ijbiomac.2022.11.295; 36464183

[17] Huang H, Zhao G, Cardenas H, Valdivia AF, Wang Y, Matei D. N6-methyladenosine RNA modifications regulate the response to platinum through nicotinamide N-methyltransferase. Mol Cancer Ther. 2023;22(3):393–405. doi:10.1158/1535-7163.MCT-22-0278; 36622754

[18] Armstrong DA, Green BB, Seigne JD, Schned AR, Marsit CJ. MicroRNA molecular profiling from matched tumor and bio-fluids in bladder cancer. Mol Cancer. 2015;14:194. doi:10.1186/s12943-015-0466-2; 26576778 PMC4650939

[19] Jin M, Shi C, Yang C, Liu J, Huang G. Upregulated circRNA ARHGAP10 predicts an unfavorable prognosis in NSCLC through regulation of the miR-150-5p/GLUT-1 axis. Mol Ther Nucleic Acids. 2019;18:219–31. doi:10.1016/j.omtn.2019.08.016; 31561126 PMC6796700

[20] Cao Y, Wang Z, Yan Y, Ji L, He J, Xuan B, et al. Enterotoxigenic *Bacteroides fragilis* promotes intestinal inflammation and malignancy by inhibiting exosome-packaged miR-149-3p. Gastroenterology. 2021;161(5):1552–66.e12. doi:10.1053/j.gastro.2021.08.003; 34371001

[21] Dai J, Su Y, Zhong S, Cong L, Liu B, Yang J, et al. Exosomes: key players in cancer and potential therapeutic strategy. Signal Transduct Target Ther. 2020;5(1):145. doi:10.1038/s41392-020-00261-0; 32759948 PMC7406508

[22] Ning T, Li J, He Y, Zhang H, Wang X, Deng T, et al. Exosomal miR-208b related with oxaliplatin resistance promotes Treg expansion in colorectal cancer. Mol Ther. 2021;29(9):2723–36. doi:10.1016/j.ymthe.2021.04.028; 33905821 PMC8417448

[23] Ding Y, Qi N, Wang K, Huang Y, Liao J, Wang H, et al. FTO facilitates lung adenocarcinoma cell progression by activating cell migration through mRNA demethylation. Onco Targets Ther. 2020;13:1461–70. doi:10.2147/OTT.S231914; 32110044 PMC7035887

[24] Song W, Yang K, Luo J, Gao Z, Gao Y. Dysregulation of USP18/FTO/PYCR1 signaling network promotes bladder cancer development and progression. Aging. 2021;13(3):3909–25. doi:10.18632/aging.202359; 33461172 PMC7906198

[25] Yu ZH, Feng ST, Zhang D, Cao XC, Yu Y, Wang X. The functions and prognostic values of m6A RNA methylation regulators in thyroid carcinoma. Cancer Cell Int. 2021;21(1):385. doi:10.1186/s12935-021-02090-9; 34281544 PMC8287668

[26] Mauer J, Luo X, Blanjoie A, Jiao X, Grozhik AV, Patil DP, et al. Reversible methylation of m6Am in the 5′ cap controls mRNA stability. Nature. 2017;541(7637):371–5. doi:10.1038/nature21022; 28002401 PMC5513158

[27] Su R, Dong L, Li C, Nachtergaele S, Wunderlich M, Qing Y, et al. R-2HG exhibits anti-tumor activity by targeting FTO/m6A/MYC/CEBPA signaling. Cell. 2018;172(1–2):90–105.e23. doi:10.1016/j.cell.2017.11.031; 29249359 PMC5766423

[28] Qing Y, Dong L, Gao L, Li C, Li Y, Han L, et al. R-2-hydroxyglutarate attenuates aerobic glycolysis in leukemia by targeting the FTO/m6A/PFKP/LDHB axis. Mol Cell. 2021;81(5):922–39. doi:10.1016/j.molcel.2020.12.026; 33434505 PMC7935770

[29] Liu J, Ren D, Du Z, Wang H, Zhang H, Jin Y. m6A demethylase FTO facilitates tumor progression in lung squamous cell carcinoma by regulating MZF1 expression. Biochem Biophys Res Commun. 2018;502(4):456–64. doi:10.1016/j.bbrc.2018.05.175; 29842885

[30] Tsai LH, Wu JY, Cheng YW, Chen CY, Sheu GT, Wu TC, et al. The MZF1/c-MYC axis mediates lung adenocarcinoma progression caused by wild-type lkb1 loss. Oncogene. 2015;34(13):1641–9. doi:10.1038/onc.2014.118; 24793789

[31] Li J, Han Y, Zhang H, Qian Z, Jia W, Gao Y, et al. The m6A demethylase FTO promotes the growth of lung cancer cells by regulating the m6A level of USP7 mRNA. Biochem Biophys Res Commun. 2019;512(3):479–85. doi:10.1016/j.bbrc.2019.03.093; 30905413

[32] Niu Y, Lin Z, Wan A, Chen H, Liang H, Sun L, et al. RNA N6-methyladenosine demethylase FTO promotes breast tumor progression through inhibiting BNIP3. Mol Cancer. 2019;18(1):46. doi:10.1186/s12943-019-1004-4; 30922314 PMC6437932

[33] Liu Y, Liang G, Xu H, Dong W, Dong Z, Qiu Z, et al. Tumors exploit FTO-mediated regulation of glycolytic metabolism to evade immune surveillance. Cell Metab. 2021;33(6):1221–33. doi:10.1016/j.cmet.2021.04.001; 33910046

[34] Tsuruta N, Tsuchihashi K, Ohmura H, Yamaguchi K, Ito M, Ariyama H, et al. RNA N6-methyladenosine demethylase FTO regulates PD-L1 expression in colon cancer cells. Biochem Biophys Res Commun. 2020;530(1):235–9. doi:10.1016/j.bbrc.2020.06.153; 32828292

[35] Lv D, Ding S, Zhong L, Tu J, Li H, Yao H, et al. M^6^A demethylase FTO-mediated downregulation of DACT1 mRNA stability promotes Wnt signaling to facilitate osteosarcoma progression. Oncogene. 2022;41(12):1727–41. doi:10.1038/s41388-022-02214-z; 35121825

[36] Sekar D, Mani P, Biruntha M, Sivagurunathan P, Karthigeyan M. Dissecting the functional role of microRNA 21 in osteosarcoma. Cancer Gene Ther. 2019;26(7–8):179–82. doi:10.1038/s41417-019-0092-z; 30905966

[37] He C, Xiong J, Xu X, Lu W, Liu L, Xiao D, et al. Functional elucidation of MiR-34 in osteosarcoma cells and primary tumor samples. Biochem Biophys Res Commun. 2009;388(1):35–40. doi:10.1016/j.bbrc.2009.07.101; 19632201

[38] Sun X, Dai G, Yu L, Hu Q, Chen J, Guo W. miR-143-3p inhibits the proliferation, migration and invasion in osteosarcoma by targeting FOSL2. Sci Rep. 2018;8(1):606. doi:10.1038/s41598-017-18739-3; 29330462 PMC5766605

[39] Jones KB, Salah Z, Del Mare S, Galasso M, Gaudio E, Nuovo GJ, et al. miRNA signatures associate with pathogenesis and progression of osteosarcoma. Cancer Res. 2012;72(7):1865–77. doi:10.1158/0008-5472.CAN-11-2663; 22350417 PMC3328547

[40] Jiao ZH, Wang JD, Wang XJ. MicroRNA-16 suppressed the invasion and migration of osteosarcoma by directly inhibiting RAB23. Eur Rev Med Pharmacol Sci. 2018;22(9):2598–605. doi:10.26355/eurrev_201805_14953; 29771408

[41] Dong C, Du Q, Wang Z, Wang Y, Wu S, Wang A. MicroRNA-665 suppressed the invasion and metastasis of osteosarcoma by directly inhibiting RAB23. Am J Transl Res. 2016;8(11):4975–81. doi:10.26355/eurrev_201805_14953; 27904698 PMC5126340

[42] Cannonier SA, Sterling JA. The role of Hedgehog signaling in tumor induced bone disease. Cancers. 2015;7(3):1658–83. doi:10.3390/cancers7030856; 26343726 PMC4586789

[43] Théry C, Zitvogel L, Amigorena S. Exosomes: composition, biogenesis and function. Nat Rev Immunol. 2002;2(8):569–79. doi:10.1038/nri855; 12154376

[44] Vlassov AV, Magdaleno S, Setterquist R, Conrad R. Exosomes: current knowledge of their composition, biological functions, and diagnostic and therapeutic potentials. Biochim Biophys Acta. 2012;1820(7):940–8. doi:10.1016/j.bbagen.2012.03.017; 22503788

[45] Shah R, Patel T, Freedman JE. Circulating extracellular vesicles in human disease. N Engl J Med. 2018;379(10):958–66. doi:10.1056/NEJMra1704286; 30184457

[46] Hannafon BN, Ding WQ. Intercellular communication by exosome-derived microRNAs in cancer. Int J Mol Sci. 2013;14(7):14240–69. doi:10.3390/ijms140714240; 23839094 PMC3742242

[47] Thakur BK, Zhang H, Becker A, Matei I, Huang Y, Costa-Silva B, et al. Double-stranded DNA in exosomes: a novel biomarker in cancer detection. Cell Res. 2014;24(6):766–9. doi:10.1038/cr.2014.44; 24710597 PMC4042169

[48] Madeo M, Colbert PL, Vermeer DW, Lucido CT, Cain JT, Vichaya EG, et al. Cancer exosomes induce tumor innervation. Nat Commun. 2018;9(1):4284. doi:10.1038/s41467-018-06640-0; 30327461 PMC6191452

[49] Jerez S, Araya H, Hevia D, Irarrázaval CE, Thaler R, van Wijnen AJ, et al. Extracellular vesicles from osteosarcoma cell lines contain miRNAs associated with cell adhesion and apoptosis. Gene. 2019;710:246–57. doi:10.1016/j.gene.2019.06.005; 31176732 PMC6684290

[50] Chang WA, Tsai MJ, Hung JY, Wu KL, Tsai YM, Huang YC, et al. miR-150-5p-containing extracellular vesicles are a new immunoregulator that favor the progression of lung cancer in hypoxic microenvironments by altering the phenotype of NK cells. Cancers. 2021;13(24):6252. doi:10.3390/cancers13246252; 34944871 PMC8699319

[51] Li H, Ouyang R, Wang Z, Zhou W, Chen H, Jiang Y, et al. MiR-150 promotes cellular metastasis in non-small cell lung cancer by targeting FOXO4. Sci Rep. 2016;6:39001. doi:10.1038/srep39001; 27976702 PMC5157020

[52] Zhao YJ, Song X, Niu L, Tang Y, Song X, Xie L. Circulating exosomal miR-150-5p and miR-99b-5p as diagnostic biomarkers for colorectal cancer. Front Oncol. 2019;9:1129. doi:10.3389/fonc.2019.01129; 31750241 PMC6842995

[53] He Z, Dang J, Song A, Cui X, Ma Z, Zhang Y. The involvement of miR-150/β-catenin axis in colorectal cancer progression. Biomed Pharmacother. 2020;121:109495. doi:10.1016/j.biopha.2019.109495; 31731194

