# CORRECTION: MiR-150-5p Inhibits Cell Proliferation and Metastasis by Targeting FTO in Osteosarcoma

**DOI:** 10.32604/or.2024.061279

**Published:** 2025-06-26

**Authors:** LICHEN XU, PAN ZHANG, GUIQI ZHANG, ZHAOLIANG SHEN, XIZHUANG BAI

**Affiliations:** 1 Dalian Medical University, Dalian, 116044, China; 2 Department of Spinal Surgery, Dalian Municipal Central Hospital, Dalian, 116033, China; 3 Department of Orthopaedics, The People’s Hospital of China Medical University, People’s Hospital of Liaoning Province, Shenyang, 110016, China; 4 Department of Orthopedic, The Third Affiliated Hospital of Jinzhou Medical University, Jinzhou, 121000, China

**Keywords:** Fat mass and obesity associated (FTO), MiR-150-5p, Oosteosarcoma (OS), Cell proliferation, Cell metastasis, exosome

In the article “MiR-150-5p inhibits cell proliferation and metastasis by targeting FTO in osteosarcoma” (Oncology Research. 2024 Oct 16;32(11):1777–1789. doi: 10.32604/or.2024.047704), an inadvertent error occurred during the compilation of Figs. 3b and 6c. This needed corrections to ensure the accuracy and integrity of the data presented.

Issue with Fig. 3b:

- Original Issue: The original Fig. 3b contained an incorrect image. Due to confusion with the folder of another NC image (Fig. 2a) during the image selection process, and due to negligence in capturing the image which led to the repeated collection of the same field of view, the image for shNC (short hairpin negative control) was incorrectly placed. This resulted in a mismatch between the image and the actual experimental conditions it was intended to depict.
- Reason for Change: To accurately represent the transwell results under the specified experimental conditions (control group), we have revised Fig. 3b. The new image correctly labels the shNC and accurately corresponds to the intended experimental setup.
- Impact on Results: The revision of Fig. 3b does not involve any alteration of the ligands or text associated with the Figure. It replaces the erroneous image with the correct one, ensuring that the visual data accurately corresponds to the reported experimental findings. This correction does not impact the scientific conclusions drawn in the study.

Issue with Fig. 6c:

- Original Issue: The original compilation of Fig. 6c contained labeling errors where the OE group was mixed with OE from Fig. 3a, and the NC group as well as the FTO+EV group were mixed with NC from Fig. 3a. These mix-ups during the labeling process resulted in images that did not accurately represent their respective experimental conditions.
- Reason for Change: To accurately depict the transwell results for the specific experimental conditions intended for Fig. 6c, we have revised the figure. The revised Fig. 6c now contains images that are correctly labeled and correspond to the intended experimental setups, without any confusion with images from other figures.
- Impact on Results: The revision of Fig. 6c solely involves the replacement of the incorrectly labeled images with the accurate ones. No alterations were made to the ligands or text associated with the figure. This change ensures that the visual data presented in Fig. 6c accurately reflects the experimental findings reported, and it does not impact the scientific conclusions drawn in the study.

The corrected versions of Figs. 3 and 6 are provided. The changes were necessary to maintain the integrity of the published work and to provide accurate visual data to support the study’s findings. We confirm that these corrections do not alter any of the study's results or conclusions, and we apologize for any inconvenience caused by the errors.

The authors would like to correct the figure as follows:

**Table:** 

Page. No.	Exact figure to be corrected	Correction
1782	Fig. 3	Replace with new Fig. 3
1785	Fig. 6	Replace with new Fig. 6

**Figure 3**

**Figure 3:**
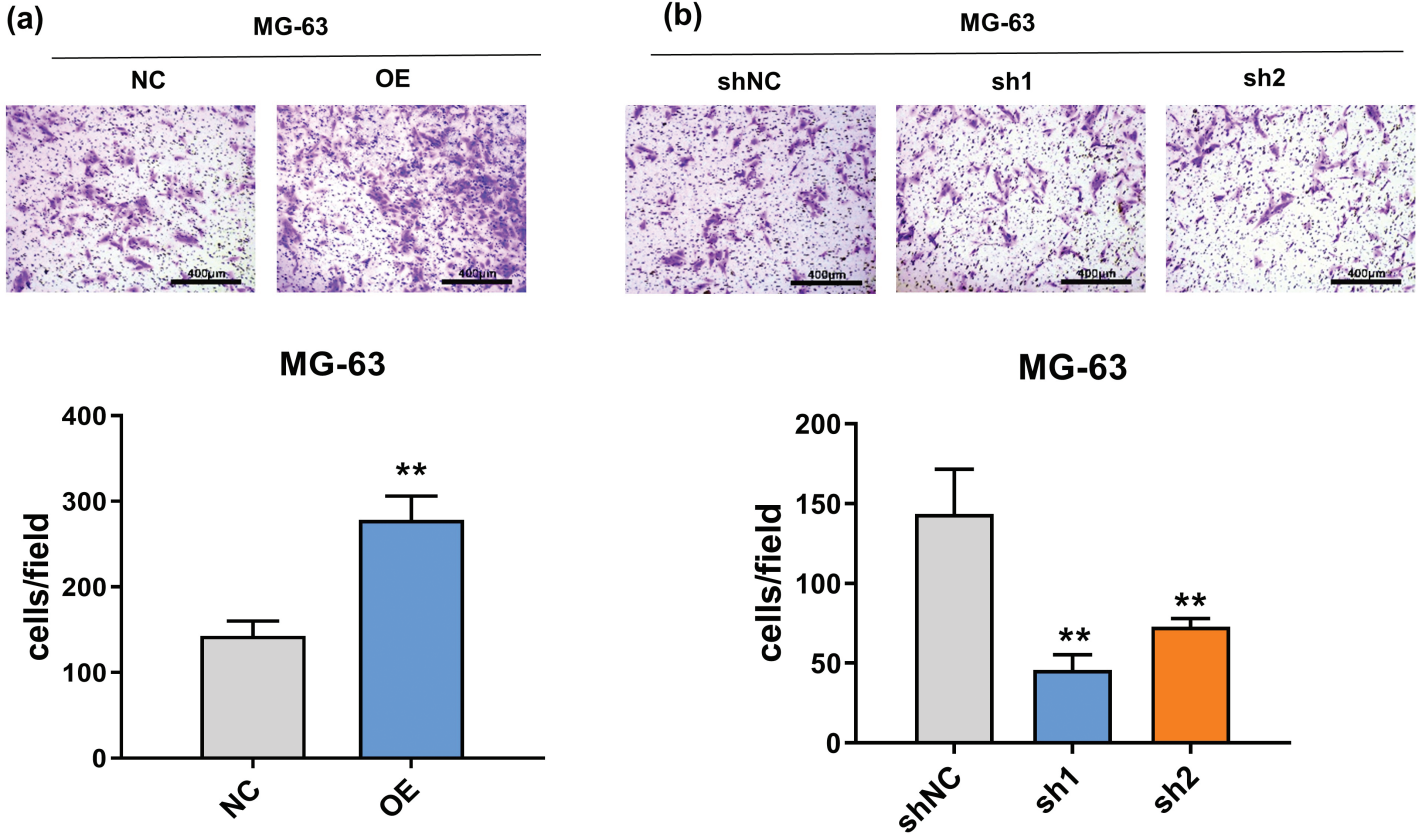
Effect of FTO on the migration of OS cells. (a, b) Transwell assay was used to detect the effect of FTO overexpression (a) or knockdown (b) on cell invasion ability. **p < 0.01, scale bar = 400 μm.

**Figure 6**

**Figure 6:**
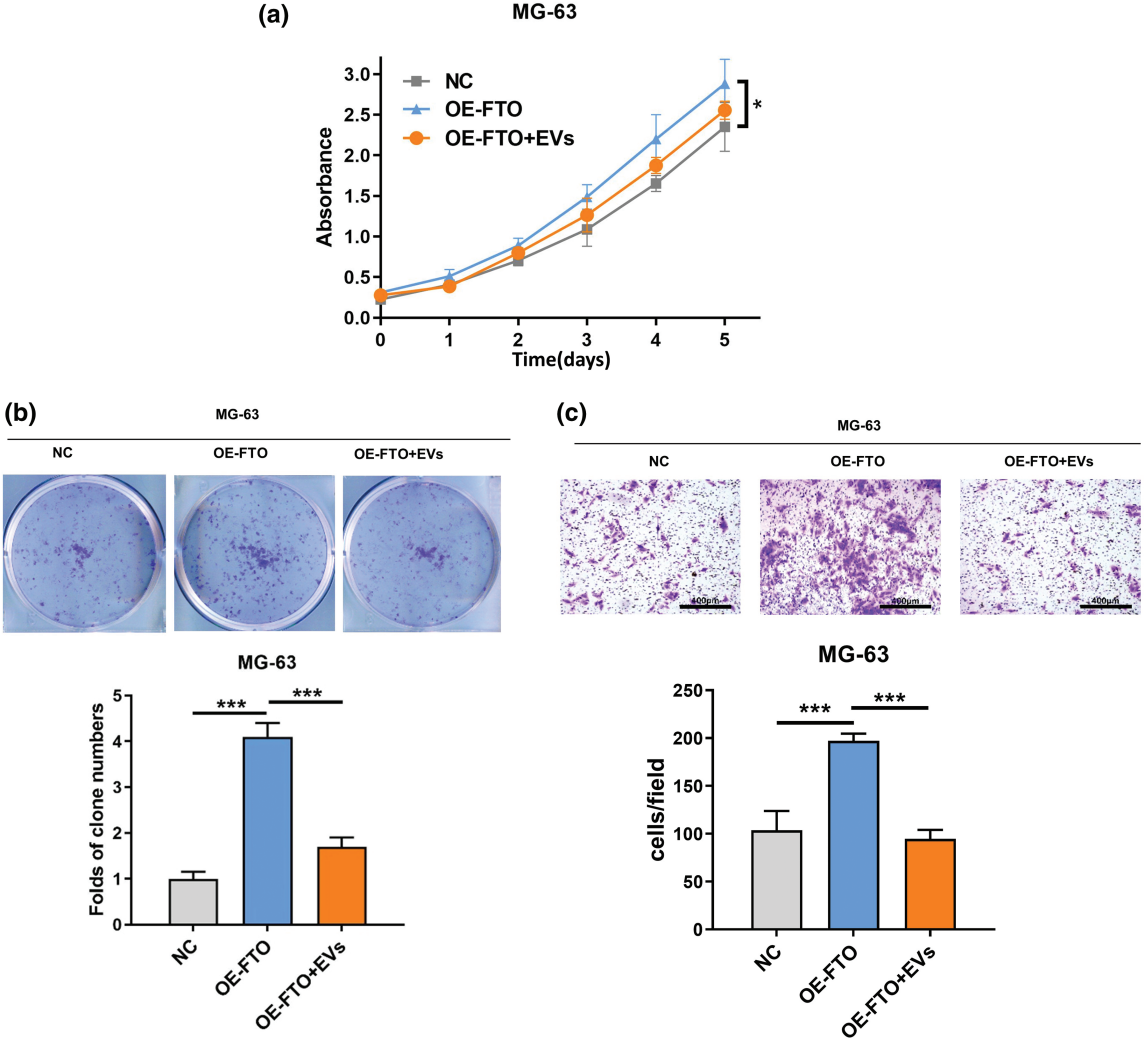
Effects of exosome miR-150-5p on proliferation and migration of OS cells overexpressing FTO. (a) CCK-8 assay. (b) Colony formation. (c) Transwell assay. *p < 0.05, ***p < 0.005, scale bar = 400 μm.

